# Innate Immune System Activation, Inflammation and Corneal Wound Healing

**DOI:** 10.3390/ijms232314933

**Published:** 2022-11-29

**Authors:** Nyemkuna Fortingo, Samuel Melnyk, Sarah H. Sutton, Mitchell A. Watsky, Wendy B. Bollag

**Affiliations:** 1 Department of Physiology, Medical College of Georgia, Augusta University, Augusta, GA 30907, USA; 2 James and Jean Culver Vision Discovery Institute, Medical College of Georgia, Augusta University, Augusta, GA 30907, USA; 3 Department of Medical Illustration, Augusta University, Augusta, GA 30907, USA; 4 Department of Cellular Biology and Anatomy, Medical College of Georgia, Augusta University, Augusta, GA 30907, USA; 5 Charlie Norwood VA Medical Center, Augusta, GA 30904, USA

**Keywords:** cornea, healing, inflammation, innate immune system, phosphatidylglycerol, toll-like receptors, wound

## Abstract

Corneal wounds resulting from injury, surgeries, or other intrusions not only cause pain, but also can predispose an individual to infection. While some inflammation may be beneficial to protect against microbial infection of wounds, the inflammatory process, if excessive, may delay corneal wound healing. An examination of the literature on the effect of inflammation on corneal wound healing suggests that manipulations that result in reductions in severe or chronic inflammation lead to better outcomes in terms of corneal clarity, thickness, and healing. However, some acute inflammation is necessary to allow efficient bacterial and fungal clearance and prevent corneal infection. This inflammation can be triggered by microbial components that activate the innate immune system through toll-like receptor (TLR) pathways. In particular, TLR2 and TLR4 activation leads to pro-inflammatory nuclear factor kappa-light-chain-enhancer of activated B cells (NFκB) activation. Similarly, endogenous molecules released from disrupted cells, known as damage-associated molecular patterns (DAMPs), can also activate TLR2, TLR4 and NFκB, with the resultant inflammation worsening the outcome of corneal wound healing. In sterile keratitis without infection, inflammation can occur though TLRs to impact corneal wound healing and reduce corneal transparency. This review demonstrates the need for acute inflammation to prevent pathogenic infiltration, while supporting the idea that a reduction in chronic and/or excessive inflammation will allow for improved wound healing.

## 1. Introduction—The Cornea

The cornea, the transparent outer layer that covers the anterior 1/6th of the eyeball, is critical for eyesight. Its importance to vision arises because this transparent (and avascular) convex tissue serves as the most powerful refractive structure in the eye, responsible, along with the lens, for focusing light on the retina. However, its position as a physical barrier to the external environment puts the cornea at risk for injury and subsequent infection. Injuries to the corneal epithelium can occur through multiple modalities including physical abrasion, burns (chemical and thermal) and even corneal surgery [1]. Corneal wounds not only cause pain but can also predispose an individual to infection. The corneal epithelial layer exhibits impressive regenerative properties, as well as mechanisms to fight microbial invasion and infection. While the cornea typically heals quickly, in some individuals, such as those with diabetes, the healing process may be delayed or impaired, resulting in significant morbidity. Delayed or impaired healing of a corneal wound and/or clearance of an infection, in turn, increases the possibility of vision impairment. Therefore, it is critically important to the individual that corneal wounds resist infection and heal rapidly.

The cornea receives nourishment anteriorly from the tear film and vessels of the limbus, and posteriorly from the aqueous humor (a fluid-filled space located behind the cornea) [2]. It consists of 5 distinct layers (from most superficial to deepest): the epithelium, which is the major focus of this review, the anterior limiting basement membrane (Bowman’s membrane), the stroma, a posterior limiting basement membrane (Descemet’s membrane) and the endothelium. Derived from surface ectoderm, the highly proliferative epithelium is the most superficial layer of the cornea and is typically 5–6 cell layers thick. It consists of a non-keratinized straightened squamous epithelium that makes up approximately 10% of the corneal thickness. The stroma constitutes about 90% of the corneal thickness and is composed mainly of connective tissue containing fibroblast-like keratocytes. The innermost single-layer endothelium is heavily involved in active ion transport, using a Na^+^/K^+^ ATPase-driven ion transport pathway to maintain the appropriate hydration state of the corneal stroma [3]. By preventing edema, the endothelial layer also helps to preserve corneal transparency [4].

According to the Centers for Disease Control (CDC), corneal diseases significantly compromise vision and negatively affect work productivity, medical and pharmaceutical costs, and quality of life. Ocular surface diseases, which include dry eye disease and pterygium as well as injuries and trauma, can even result in blindness. Corneal pathologies are the 4th leading cause of blindness and affect the productivity and quality of life of nearly 5 million people worldwide [5]. Additionally, the financial cost associated with ocular surface disease has been an increasing burden on both the US and global economies. For example, the overall burden of dry eye disease in the US healthcare system is calculated at almost 4 billion dollars (from the payer’s perspective) and more than 55 billion dollars to society, although the true overall cost may be even higher [6]. Direct costs to patients increase in correlation with decreasing visual acuity. The annual direct medical cost per patient for those with mild, moderate, and severe dry eye disease symptoms is estimated at about 680, 770, and 1270 dollars, respectively [6]. Veterans, in particular, can be susceptible to dry eye disease as a result of their exposure to the extreme environmental conditions often found in combat areas [7], which can result in corneal abrasions and pain [8,9], affecting vision. Indeed, a 2013 study found that almost half of male veterans 50 years or older attending an eye care clinic reported symptoms of severe dry eye disease [9]. A subsequent study in Operation Iraqi Freedom and Operation Enduring Freedom veterans found that more than a quarter of these younger veterans also reported severe dry eye symptoms [7,10], suggesting the significance of this syndrome in the veteran population. A better understanding of the mechanisms underlying corneal wound healing, such as the corneal abrasions arising from dry eye disease, as well as of the dysregulation of the wound healing process that occurs in individuals with impaired healing, should allow for development of treatments that accelerate corneal healing, which will be beneficial to patients with these very painful and potentially vision-compromising wounds.

## 2. Corneal Wound Healing

### 2.1. The Healing Process

Corneal epithelial wound healing is a complex multistep process involving apoptosis, cellular migration, proliferation, differentiation, and extracellular matrix interactions at the wound site [11]. There are four overlapping phases associated with corneal wound healing: the latent or lag phase, followed by the migration, proliferation and attachment/adhesion phases, which lead to wound closure. The initial latent stage involves cellular reorganization, such as hemidesmosomal detachment and subcellular actin polymerization to allow migration to the wound site [2]. Once cells become motile, wound closure is initiated as a result of increased cellular migration. Proliferation of the cells allows restoration of corneal thickness through stratification. There is then an enhanced generation of hemidesmosomes to help anchor the tissue to the basement membrane [12]. Immune cells infiltrate the cornea beginning late in the latent phase, and the resultant inflammatory response clears damaged cells and bacteria, in part mediated by the stimulation of a Th1 immune response, leading to fibrocyte innate immune functions that further contribute to the wound healing process [13].

The latent phase, consisting of cell detachment from the basement membrane and cytoskeletal restructuring, gives time for the wound to be prepared for the healing process. Here, epithelial cells in the unaffected region begin the process of flattening to eventually migrate across the open wound area and form a thin sheet that seals the wound. In addition, integrins, transmembrane proteins necessary for the linkage of cytoskeletal components to the basement membrane, mediating several adhesive functions and triggering signal transduction pathways into the cell (i.e., outside-in signaling), dissociate and redistribute along the cell surface [14]. In the later stages of this phase, immune cells begin to infiltrate into the cornea and initiate an immune/inflammatory response.

**Figure 1:**
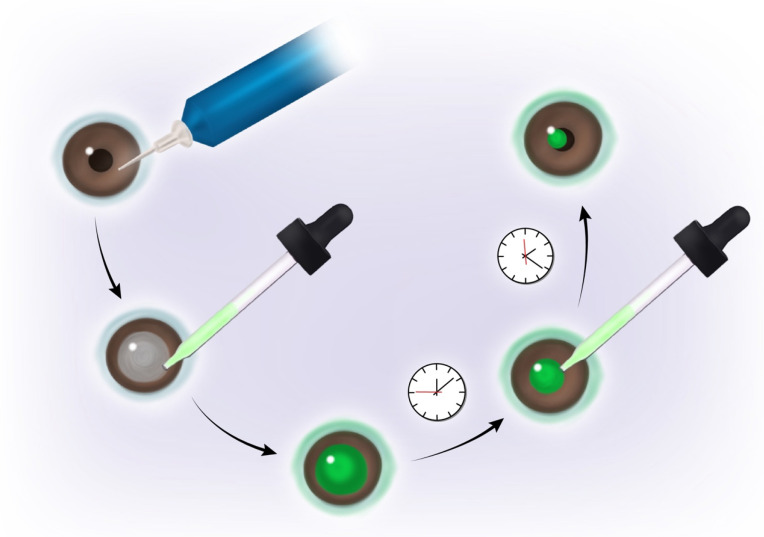
Monitoring Corneal Wound Healing. Corneal epithelial wounds are created with an Alger brush, which is used to scrape off the corneal epithelium. Exposure of the underlying stroma allows visualization of the wound with the dye fluorescein, which binds to the stroma but not the intact epithelium. The rate of wound healing can be monitored over time by measuring the percentage of the wound area remaining as visualized with fluorescein. Agents of interest can be topically applied to the corneal surface (once or repeatedly) to determine their impact on corneal wound healing.

The latent phase then proceeds into migration and re-epithelialization [14]. In this case, a thin sheet of protective epithelial cells is formed, via migration of the corneal epithelial cells across the wound largely as a coherent sheet, to cover and close the open wound area and begin to re-establish the structure of the corneal epithelium (re-epithelialization). The process of wound closure can be monitored as illustrated in Figure 1. During this phase there is rearrangement of actin-rich stress fibers that are necessary for supporting the migratory processes that restore the wounded corneal epithelium. Lastly, proliferation, stratification, and differentiation restore the corneal epithelial cell layers; the cells on the basement membrane then form permanent hemidesomosomal attachments to complete the healing process and anchor the epithelium to the underlying connective tissue. Growth factors, such as epidermal growth factor (EGF), keratinocyte growth factor 1 (KGF-1), and hepatocyte growth factor (HGF) can stimulate cellular migration and/or proliferation, thus allowing for more rapid corneal wound healing [14]. Upon injury at the epithelial-stromal base, these growth factors enter through the damaged epithelium into the stroma, triggering a response from myofibroblasts, which under certain conditions, can survive and proliferate to result in a corneal haze [15].

### 2.2. Importance of the Limbal Stem Cell Population and Certain Signaling Pathways

In terms of the corneal epithelium, research has shown that limbal stem cells (LSCs) serve as the cell reservoir with the proliferative capacity needed to regenerate the cells required for wound healing. The LSCs, with essentially unlimited proliferative capacity, give rise to a more limited transit amplifying cell that is destined to become a post-mitotic cell that terminally differentiates and migrates from the limbus to the central cornea [16] and/or wound site. Arrested differentiation coupled with the slow cell turnover rate and high proliferative capacity of the LSCs allows for regeneration of the corneal epithelium while maintaining corneal homeostasis [17]. LSC deficiency (LSCD) is a major concern due to its association with an increased risk of blindness, since reduced LSC-mediated corneal epithelial repair can lead to the loss of healthy epithelium with resultant corneal inflammation and vascularization [18]. LSCs are marked by expression of the ATP-binding cassette, sub-family B, member 5 (ABCB5). Initially identified in skin progenitor cells and melanoma, ABCB5 serves as a regulator of stem cell differentiation necessary for LSC maintenance and therefore corneal repair, and its absence leads to increased corneal opacity and LSCD-like epithelial conjunctivalization [19].

Upon injury, the corneal epithelial cells must be mobilized to restore and then regenerate the epithelial structure. The series of cellular processes needed to repair the cornea are initiated in response to various cell signals in order to restore corneal homeostasis. Growth factors produced in response to corneal epithelial injury, including EGF and cytokines like interleukin (IL)-1 (IL-1), regulate the migration, proliferation, differentiation and stratification that occur during wound healing [20]. Several cytokines also have pro-inflammatory activity and seem to play a role in the wound healing process in part by activating the innate immune system to protect against microbial infection. Others have different roles, contributing to a cascade of intercellular responses that are necessary for the coordination of corneal wound healing, such as orchestrating the appropriate production of growth factors [20]. The initial set of responses is rapid and well-regulated and depends on the activation of the EGF receptor (EGFR), as well as the response to IL-1 and tumor necrosis factor-alpha (TNFα), which play crucial roles in mediating corneal wound healing as well as initiating inflammation [20] (see below).

For example, EGF is well-known for its wound healing properties, with repeated demonstrations of its ability to promote corneal epithelial wound healing. EGF enhances cellular migration, proliferation, and differentiation of corneal epithelial cells, and its use in vivo has been shown to increase rates of corneal epithelial wound healing. When EGF binds to its receptor EGFR, the receptor dimerizes, leading to the activation of its endogenous tyrosine kinase activity and its autophosphorylation [21]. Indeed, EGFR activation/phosphorylation is necessary for corneal epithelial wound healing, as inhibition of this activation leads to impaired wound healing [22], at least in part due to reduced cellular migration [23]. Although EGFR activation is critical for corneal epithelial wound healing, it should be noted that there are multiple additional ligands that can activate the receptor, including amphiregulin, heparin-binding-EGF (HB-EGF) and transforming growth factor-alpha (TGFα). The fact that these EGFR ligands are expressed by corneal epithelial cells [22] suggests that these agents might also contribute to corneal wound healing. However, Peterson et al. [21] found that although epiregulin, betacellulin, TGFα, HB-EGF, amphiregulin and EGF were all detectable in human tear fluid (from *unwounded* eyes), only EGF was at a concentration near its reported K_d_. Similarly, although all of the EGFR ligands (except epiregulin, which was not tested) accelerate corneal epithelial wound healing in vitro, with some (betacellulin and HB-EGF) showing greater efficacy than EGF, only EGF improves corneal wound healing in mice in vivo. On the other hand, others have demonstrated an ability of endogenous and exogenous HB-EGF to promote corneal epithelial wound healing in vitro and in porcine organ cultures [22], as well as to increase release of amphiregulin upon corneal epithelial cell wounding [24]. Finally, it should be noted that the EGFR can also be transactivated by certain G-protein-coupled receptors, and in fact, Block and Klarlund [24] have shown the ability of ATP released from damaged corneal limbal epithelial cells to transactivate the EGFR through purinergic receptors. Thus, the EGFR and its ligands appear to be important players in corneal wound healing. However, less clear is the role of cytokines/chemokines released by innate immune cells in this process. A great deal of evidence supports the idea that activation of the innate immune system and the resultant inflammation can assist in the clearance of a microbial infection as will be described in the next section. On the other hand, whether or not inflammatory factors can also promote corneal wound healing is less obvious, although evidence is accumulating that some inflammation is beneficial for this process, as discussed below.

### 2.3. Importance of the Innate Immune System and Cytokines in Wound Healing and Corneal Inflammation

**Figure 2:**
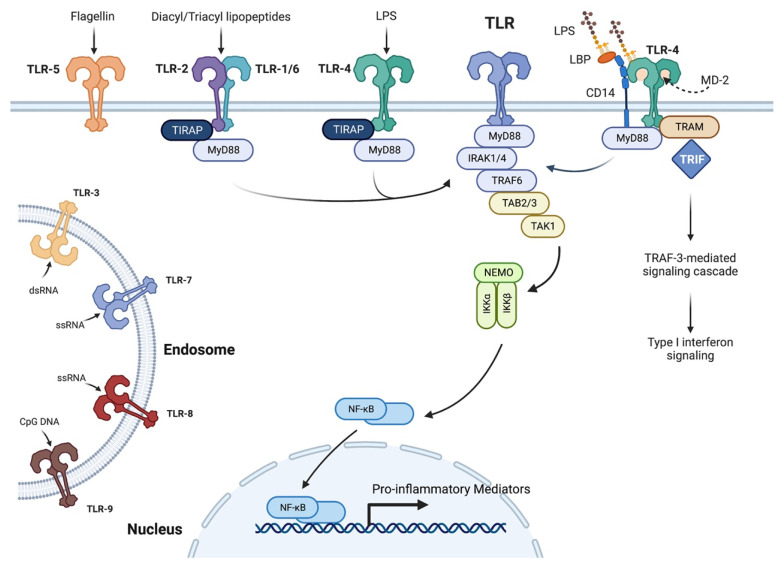
Toll-like Receptor Signaling Pathway. Toll-like receptors are located in the plasma membrane (TLR-2/-1, TLR-2/-6, TLR-4 and TLR-5) or the endosome (TLR-3, TLR-7, TLR-8 and TLR-9) where they are engaged by the indicated microbial components to initiate signaling through myeloid differentiation primary response 88 (MyD88) and in some cases the adaptor TIR domain-containing adaptor protein (TIRAP). MyD88 interaction with interleukin-1 receptor-associated kinase (IRAK) protein kinases activates TNF receptor-associated factor-6 (TRAF6) and through the adaptors TAB2 and 3, the kinase (TGFβ)-activated kinase 1 (TAK1). TAK1 phosphorylates and activates inhibitory kappaB (IκB) kinases (IKK)-α and -β, which are in complex with a regulatory subunit, NFκB essential modulator (NEMO), also known as IKKγ. Phosphorylation of IκB targets it for degradation thereby releasing the transcription factor, nuclear factor kappa-light-chain-enhancer of activated B cells (NFκB). NFκB translocates into the nucleus where it promotes the expression of pro-inflammatory mediators. TLR-4 signaling also involves lipopolysaccharide (LPS)-binding protein (LBP), which helps to present LPS to the TLR-4 receptor complex, which also includes myeloid differentiation protein-2 (MD-2) and the co-receptor cluster of differentiation-14 (CD-14). LPS binding to TLR-4 can also activate a MyD88-independent pathway through TIR domain-containing adaptor inducing interferon-β (TRIF) and translocation-associated membrane protein (TRAM) to activate TRAF3 to induce type I interferon expression; dsRNA = double-stranded RNA, ssRNA = single-stranded RNA and CpG DNA = CpG-rich DNA. Created with Biorender.com.

The innate immune system is the first line of defense against invaders and is responsible for protecting from external threats such as bacteria, viruses, and fungi. The innate immune system makes use of pattern recognition receptors (PRRs) that respond to pathogen-associated molecular patterns (PAMPs), which are microbial components like lipopolysaccharide (LPS), that initiate an immune response [25]. The most well-characterized and well-known PRRs are the toll-like receptors (TLRs). TLRs are trans-membrane proteins that, upon binding of a PAMP, trigger a downstream pathway to initiate an immune response [25]. Each TLR has a specific PAMP that it recognizes (Figure 2): for example, TLR4 recognizes lipolysaccharide (LPS). Other TLRs include: TLR2, which forms a complex with either TLR1 or TLR6, recognizing lipoproteins; TLR7 and TRL8 recognizing single-stranded RNA; and TLR9 recognizing CpG sequences found in bacterial and viral DNA [25]. Finally, TLR3 recognizes double-stranded RNA and TLR5, bacterial flagellin, and TLR11 and TLR12 recognize parasite profilin (the TLR10 ligand is unknown at this time).

All TLRs, except TLR3, use myeloid differentiation primary response 88 (MyD88), an adaptor protein, to help transmit signals, resulting in the activation of the transcription factor, nuclear factor kappa-light-chain-enhancer of activated B cells (NFκB), which induces the production of pro-inflammatory cytokines [25]. Generally, subsequent to MyD88 activation, the activity of interleukin-1 receptor-associated kinases (IRAK) is stimulated, which phosphorylate and activate TNF receptor-associated factor-6 (TRAF6). TRAF6 then activates transforming growth factor-beta (TGFβ)-activated kinase 1 (TAK1), which phosphorylates and activates the inhibitory kappaB (IκB) kinase (IKK) complex to phosphorylate IκBs, resulting in the release of NFκB. NFκB then translocates into the nucleus to initiate production of inflammatory cytokines such as IL-1 [25]. This signaling pathway can also activate mitogen-activated protein kinase (MAPK) cascades that phosphorylate and activate the transcription factor, activating protein-1 (AP-1). While most TLRs use MyD88 as an adaptor protein, TLR2 and TLR4 also use TIR domain-containing adaptor protein (TIRAP) to help recruit MyD88. As illustrated in Figure 2, TLR4′s recognition of LPS is facilitated by LPS-binding protein (LBP), as well as the adaptor/co-receptor proteins, cluster of differentiation-14 (CD14) and myeloid differentiation protein-2 (MD-2). LPS directly binds to LBP, which then transfers the LPS to CD14; CD14 in turn is thought to shuttle LPS to the MD-2/TLR4 complex, which undergoes a change in conformation upon LPS binding.

CD-14 also appears to serve as a co-receptor for TLR2 [26,27]. The conformational change in the MD-2/TLR4 complex induced by LPS binding is then detected by the adaptor proteins MyD88 and toll-like receptor adaptor molecule-1 (TICAM1, also known as TRIF), eventually triggering two separate signaling pathways [25,28]; initially the MyD88 pathway is activated, but subsequently, TLR4 is endocytosed to form a complex with translocation- associated membrane protein (TRAM) and TRIF. This complex then recruits TRAF3 to initiate a signaling pathway that leads to the expression of type I interferons. TRAM-TRIF also recruits TAK1 leading to a late phase activation of NFκB as well as mitogen-activated protein kinase pathways [25,28]. The innate immune response is necessary for defense against many infections, and evidence suggests that some cytokines, such as IL-1α and β, can promote wound healing in addition to recruiting immune cells into the cornea and promoting an inflammatory response.

#### 2.3.1. Role of IL-1 in Wound Healing and Corneal Inflammation

IL-1, which includes both IL-1α and IL-1β, regulates multiple processes in corneal wound healing, including through direct effects on corneal keratocytes involved in wound healing. For example, IL-1 modulates the regulated cell death of keratocytes (see below). Moreover, IL-1 mediates various aspects of the wound healing response, including neovascularization, chemotaxis, and stimulation of immune cells [29]. IL-1 has been shown to also stimulate corneal epithelial cells to produce β-defensin-2 [20], an anti-microbial peptide that protects the tissue from microorganisms but may also act as a damage-associated molecular pattern (DAMP) to activate TLRs [30,31] and amplify inflammation.

IL-1α and IL-1β are released from corneal epithelial cells upon cleavage of their precursor proteins by caspase-1 [20]. Both IL-1α and IL-1β serve the dual purpose of being pro-inflammatory as well as pro-healing. They are important modulators of the inflammatory cell response to corneal injury as well as a defense against infectious pathogens [20]. For example, IL-1α and IL-1β increase the apoptotic activity of keratocytes through the Fas-Fas ligand system [32]. This can promote healing of corneal wounds by inhibiting microbial spread through induction of the death of infected keratocytes [20], thereby proactively removing keratocytes that might serve as a vector of spread. Similarly, IL-1, together with transforming growth factor-β (TGF-β), modulates myofibroblast survival [20]. Thus, a wound-induced increase in TGF-β promotes myofibroblast generation, but when TGF-β levels are reduced upon healing of the wound, IL-1 triggers the myofibroblast apoptosis and keratocyte repopulation that is important for restoring corneal transparency [20]. IL-1 also regulates the production of growth factors (and cytokines/chemokines) involved in restoration of proper corneal structure [20]. Finally, IL-1α and IL-1β are known for their pro-inflammatory actions, with increased expression of IL-1 correlating with increased inflammation in the cornea. IL-1 can activate inflammatory and antigen-presenting cells [29], which can then act on surviving keratocytes to promote the upregulation of chemokine production that recruits immune cells into the cornea. Indeed, immune cell infiltration into the cornea after corneal wounding is reduced upon treatment with an inhibitor of IL-1′s action, IL-1 receptor antagonist (IL-1RA), indicating the importance of IL-1 to this process [20].

#### 2.3.2. Role of TNF-α and IL-6 in Corneal Inflammation

TNFα is another well-known cytokine that is widely recognized for its broad inflammatory effects in various cell types. Produced by neutrophils, activated lymphocytes, macrophages, and natural killer cells, TNFα possesses proapoptotic capabilities [33]. TNFα is also able to activate the NFκB pathway involved in inflammatory responses [33]; NFκB has been shown to be required for retaining corneal transparency upon exposure of the eye to ultraviolet light [34]. TNFα also inhibits the action of vascular endothelial growth factor (VEGF) and TGF-β to suppress neovascularization of the wounded cornea, such that genetically manipulated mice lacking TNFα exhibit greater stromal neovascularization upon wounding [35]. Similarly, IL-6 is another pro-inflammatory cytokine. When the IL-6 receptor bound to IL-6 comes into contact with the GP130 signaling complex, Janus kinase (JAK) is phosphorylated and activated, leading to signal transducer and activator of transcription-3 (STAT3) activation [20,36]. As a result of STAT3 activation, there is increased inflammation and apoptosis. Prior studies have shown that with chronic ocular inflammation, the IL-6 receptor is upregulated, thus allowing for a continued, i.e., chronic, immune response [36].

## 3. Injury-Induced Corneal Inflammation and Its Role in Wound Healing

As discussed above, the cornea serves as the first-line defense of the eye to environmental insults; in fact, because it interfaces with the external environment, the cornea is extremely susceptible to injury. Corneal wounds as a result of such injuries, as well as from chemical burns, surgery and contact lens wear, are not only painful but also predispose individuals to corneal infection and keratitis. Indeed, during the course of human evolution, the possibility of infection was likely the most pressing issue in terms of the health and survival of the individual, since infection can lead to blindness, which reduces fitness and thus threatens the organism’s existence. Therefore, to combat potential infection, wounding induces activation of the immune system, in particular, the innate immune system, through stimulating PRRs like the TLRs, as discussed earlier. The activation of the innate immune system in response to corneal injury should help to prevent and/or oppose infectious keratitis, which could develop as a result of the compromised corneal barrier allowing entry of pathogens into the cornea and/or the eye itself (i.e., the globe). Activation of the innate immune system results in the production of various chemokines and cytokines to enhance microbial clearance of the ocular surface, thereby inhibiting infection and promoting a more positive outcome, as discussed below.

### 3.1. Infectious Corneal Inflammation (Nonsterile)

As previously mentioned, corneal injury can increase the risk of infections, which can potentially impact eyesight. Indeed, infectious keratitis can present as a vision-threatening ocular emergency, which if untreated, can lead to visual impairment and blindness. This disorder disproportionately affects marginalized groups and developing countries. In the United States, infectious keratitis is associated with contact lens use [37] and can be due to bacterial and fungal pathogens, as described below, as well as viruses, such as herpes virus, adenovirus and recently SARS-CoV-2 (the virus causing COID-19); characteristics of these various viral corneal infections were recently reviewed in references [38,39,40] and will not be addressed here, although similar innate immune system mechanisms can be activated by viral PAMPs. In subsequent sections, common bacterial and fungal infectious agents will be identified and the role of the innate immune system in responding to these agents will be described.

#### 3.1.1. Bacterial Infection

Some of the most common pathogens that are associated with bacterial keratitis include: *Staphylococcus aureus, Staphylococcus epidermidis, Streptococcus pneumoniae*, and *Pseudomonas aeruginosa* [41]. Gram-positive organisms, such as coagulase-negative *Staphylococcus,* (e.g., *Staphylococcus epidermidis)*, are more common causes of infection than Gram-negative bacteria. However, a common Gram-negative strain causing microbial keratitis is *Pseudomonas*, which is often associated with prolonged contact lens wear [42].

##### *Pseudomonas aeruginosa* 

*Pseudomonas aeruginosa* is the most frequent cause of keratitis caused by Gram-negative bacteria and the most pathogenic of the commonly involved bacterial species [43]. The first step by which these microbes cause infection of the corneal surface is through attachment to its surface via adhesins, often found on the pili and fimbriae [44]. In turn, adhesins serve as exogenous toxins that activate an immune cascade in response to the initiation of the microbial infection. Like all Gram-negative bacteria, *P. aeruginosa* produces LPS, which activates the innate immune system through activation of TLR4, as discussed above. TLR4, again like all TLR receptors except TLR3, signals though TIRAP to recruit MyD88 [45], also as discussed above. The resulting activation of this pathway leads to the translocation to the nucleus and activation of NFκB and the infiltration of neutrophils into the corneal stroma. The attendant inflammation results in increased bacterial clearance but can also cause tissue damage [45]. In particular, TLR4 activation leads to the secretion of proinflammatory cytokines, such as IL-1β, macrophage inflammatory protein (MIP)-2, IL-6, IL-12, IL-18, and interferon (IFN)-γ [46]. An absence or deficiency of TLR4 in mice results in increased susceptibility to *P. aeruginosa* infection compared to wild-type mice that are normally resistant to the microorganism. In these TLR4-deficient mice, an inhibition of the upregulation of pro-inflammatory cytokines and antimicrobial products, such as β-defensin-2, is accompanied by a decrease in bacterial killing and reduced clearance [46,47]. Thus, the activation of the TLR4 pathway appears to serve as a protective means to prevent infection, through its upregulation of inflammatory pathways.

*P. aeruginosa* flagellin can also activate TLR5 on corneal stromal macrophages [48] to trigger the release of pro-inflammatory mediators and promote neutrophil recruitment, actions that also lead to the killing and clearance of the bacteria. One study demonstrated that pretreatment of corneal epithelial cells with flagellin results in activation of a protective response, with suppression of immune cell infiltration and increased bacterial destruction and clearance during the early stages of infection, and a decrease in polymorphonuclear (PMN) leukocytes during the late stages of bacterial infection. Moreover, there is a reduction in tissue damage due to the decrease in the late-stage inflammatory response and suppression of pro-inflammatory cytokines. Since flagellin pretreatment inhibits immune cell infiltration and inflammation, these results suggest that the innate immune response can be excessive in terms of what is necessary to clear certain microorganisms, and detrimental in terms of tissue damage, such that suppression of inflammation, especially during late-stage infection, produces a better outcome [49].

As noted above, while induction of pro-inflammatory cytokines serves a beneficial role to protect the corneal epithelium in the face of infection, too much inflammation and/or chronic inflammation produces negative effects. For example, high mobility group box 1 (HMGB1) is a protein released by damaged cells during infection (or sterile inflammation, see below) to serve as a DAMP and late-stage proinflammatory mediator. It is composed of three domains, Box A–C. Box A has been shown to be an HMGB1 antagonist, and corneal application of Box A reduces both bacterial load and neutrophil infiltration [50], suggesting that this protein may represent a potential therapeutic option to reduce proinflammatory cytokines in the *P. aeruginosa*-infected cornea. This positive outcome for infectious keratitis occurs in the absence of a strong and/or chronic inflammatory response. Finally, another study demonstrated that siRNA treatment to reduce the levels of TLR9, a pattern recognition receptor activated by unmethylated CpG sequences found in bacterial and viral DNA, also leads to better corneal outcomes [51], again suggesting that too much inflammation is likely detrimental for optimal corneal wound healing.

##### *Staphylococcus aureus* 

*Staphylococcus aureus*, a Gram-positive, nonmotile bacteria, has also been observed to be a common pathogen in infectious ocular disease. The α-toxin secreted by this microorganism has the ability to generate an immune response leading to damage to the ocular surface [52]. In response to acute *S. aureus* infection, there is an upregulation of chemokine production and neutrophil recruitment to the corneal stroma. As a result, neutrophils degranulate to release “cytotoxic mediators”, which cause tissue damage and increased corneal opacity [53]. Nevertheless, stimulation of an innate immune response by pathogen-mediated TLR2 activation serves as a primary defense against *Staphylococcus aureus*-induced keratitis. As a result of the initial innate immune system activation, there is increased clearance of the bacteria. Then, increased differentiation of keratocytes into myofibroblasts (induced by TLR2 signaling), along with the increased production of chemokines, serves as a secondary defense [54]. Thus, some inflammation is required as a proper response to infection, because some immune system activation is necessary to promote corneal cell survival and microbial clearance and thus decrease susceptibility to *S. aureus* infection [55]. Nevertheless, excessive and/or chronic inflammation can lead to tissue damage, as noted above.

#### 3.1.2. Fungal Infection

Mycotic keratitis is an opportunistic fungal infection of the cornea often resulting from injury of or trauma to the corneal epithelium, although immunocompromise can also increase the risk of developing this infection. Without proper diagnosis and treatment, mycotic keratitis can have a poor prognosis and lead to vision loss and possible corneal perforation [56]. Typically, mycotic keratitis is due to filamentous fungi, with the most common fungal corneal pathogens being *Aspergillus* [57] and *Fusarium* [56]. Once the epithelial barrier is compromised through physical trauma, the fungus can infiltrate the corneal epithelial meshwork. Typically, once the fungal pathogen enters, it is recognized by a pattern recognition receptor, which leads to activation of the innate immune system. Subsequently, there is recruitment of neutrophils, monocytes and macrophages and then a subsequent adaptive immune response. However, a reduced/inhibited innate immune response can lead to continued infection [58]. The innate immune response to the pathogenic infiltration of the corneal epithelium by fungi is mediated by TLR2 and TLR4 [59].

##### *Aspergillus* 

*Aspergillus* is a filamentous fungus that plays a crucial role in fungal keratitis around the world. The main subspecies that causes Aspergillus keratitis in developed and developing countries is *Aspergillus fumigatus*, which is strongly associated with aspergillosis, an extremely invasive fungal infection that profoundly affects immunocompromised individuals. Upon infection by *A. fumigatus*, TLR4 is upregulated. A recent study [60] demonstrated a strong association between lectin-like oxidized-LDL receptor (LOX-1), TLR4, and the upregulation of reactive oxygen species (ROS), a direct attempt of the immune system to kill the fungus in response to infection. Indeed, LOX-1 expression is upregulated in *A. fumigatus*-infected mouse corneas, suggesting its possible role in the initial innate immune response. On the other hand, excessive accumulation of ROS can have damaging effects on various pathways and on the cornea itself. Together, both LOX-1 and TLR4 increase ROS generation and when either is inhibited/reduced, ROS generation is decreased, with a lower neutrophil count and reduced inflammation [60]. For example, LOX-1 inhibition results in a reduced pro-inflammatory response to DAMPs such as high-mobility group box 1 (HMGB1) in *A. fumigatus* keratitis [61]. HMGB1 acts as a proinflammatory mediator through its ability to activate TLR4 [62], exacerbating corneal inflammation in *A. fumigatus* keratitis via this pathway [62]. In addition, upon pretreatment with BoxB of HMGB1 in the *A. fumigatus*-infected cornea there is an enhanced upregulation of LOX-1. However, when LOX-1 is inhibited, the induction of several proinflammatory cytokines, including TNFα, C-X-C motif chemokine ligand-1 (CXCL1), matrix metalloproteinase-9 (MMP9) and IL-6, is decreased [63]. Rapamycin also downregulates TLR4 and the proinflammatory cytokine IL-1β during *Aspergillus fumigatus* keratitis and exerts a beneficial effect to reduce disease severity, demonstrating that excessive inflammation produces negative outcomes; nevertheless, a certain degree of inflammation is necessary for proper fungal clearance [64].

TLR2 activation also upregulates pro-inflammatory cytokines, and therefore, corneal inflammation, as well as tissue damage in rats with *Aspergillus fumigatus* keratitis. siRNA-mediated knockdown of TLR2 not only improves disease outcomes but also protects corneal tissue from more severe damage in comparison to the control siRNA [65]. The suppressed inflammatory response is beneficial in that there is improved corneal transparency, reduced instances of corneal perforation, inhibited infiltration of polymorphonuclear (PMN) cells, decreased levels of pro-inflammatory cytokines/chemokines, and increased fungal clearance [65]. NOD2 also induces inflammatory pathways through activation of innate immunity in corneal epithelium exposed to *A. fumigatus* conidia (the fungal spore). Similar to the effects observed with TLR2, NOD2-specific siRNA also reduces levels of pro-inflammatory cytokines [66].

As previously mentioned, excessive inflammation can lead to severe tissue damage. Activation of triggering receptor expressed on myeloid cells-1 (TREM-1), which has been shown to increase the production of pro-inflammatory cytokines like TNFα and IL-1β, can lead to excessive inflammation [67]. TREM-1 plays a role in regulating TLR2 and TLR4, enhancing their respective activation in *Aspergillus fumigatus* infection and thus amplifying corneal inflammation [67]. This same pattern has also been observed in *P. aeruginosa* infection through modulation of TLR signaling and Th1/Th2 immune responses [68]. Tacrolimus, an immunosuppressant, has been shown to reduce the severity of corneal damage in earlier stages of *A. fumigatus* fungal keratitis by downregulating TREM-1 [67]. Finally, TREM-1 works synergistically with Dectin-1 to amplify the infiltration of neutrophils, macrophages, and dendritic cells and the production of proinflammatory cytokines like TNFα, which in turn leads to excessive inflammation and subsequent tissue damage. Inhibition of TREM-1 and Dectin-1 prevents excess inflammation by suppressing the secretion of pro-inflammatory cytokines, thus improving the outcomes of corneal wound healing [69].

##### *Fusarium* 

*Fusarium*, a genus of filamentary fungus, is the most common cause of fungal keratitis in parts of the southern United States, Africa and Asia [70,71]. Typically, this species is found in hot and humid areas with a great deal of agricultural activity. Members of the family include *Fusarium solani* and *Fusarium oxysporum,* which can survive as plant pathogens and saprophytes. *Fusarium* species can be the cause of systemic fusariosis and, along with other filamentous fungi like *Aspergillus*, endophthalmitis in immunocompromised individuals. However, the usual result of *Fusarium* infection is fungal keratitis, which occurs in otherwise healthy people engaged in agricultural work [70] or in healthy young individuals, often due to an ocular trauma or contact lens wear [71], in contrast to *Aspergillus*. Indeed, in the USA, of the 318 reported cases of ocular *Fusarium* in 2006, 94% of the cases were associated with soft contact lens wear. Studies have shown that *Fusarium* can attach to and infiltrate soft contact lenses, forming a biofilm layer that can be structurally different depending on the contact lens type. Once this biofilm has been formed, it becomes more difficult for interventional measures such as antimycotics to exert their effects [59].

A study examining *F. oxysporum* grown as a biofilm on soft contact lenses demonstrated the induction of keratitis following ocular trauma. The innate immune system-mediated response involves IL-1 and TLR4 and their effector, MyD88. Indeed, TLR4 plays a significant role in fungal killing because in TLR4 knockout mice, there is impaired fungal clearance and increased corneal disease [70]. However, interestingly, TLR4 does not seem to play a role in the observed corneal opacification, because the TLR4 knockout mice show similar corneal opacity to the wild-type C57BL/6 mice [59]. The importance of the TLR4 pathway has been further corroborated by another study showing that MyD88 is essential for resolution of *Fusarium*-dependent fungal keratitis. According to this study, fungal killing is directly dependent on TLR4, with TLR2 playing no significant role. Thus, a certain degree of an innate immune response through TLR4 is necessary to reduce fungal load following ocular trauma and invasion of pathogenic species.

More recent studies show that TLR2 also plays a role in *F. solu* infection, through its induction of IL-10 to produce an anti-inflammatory resolution response. Both the *F. solu* hyphae and conidia are able to stimulate telomerase-immortalized human stromal fibroblasts to increase expression of IL-1β and IL-10. Interestingly, both IL-1β and IL-10 function simultaneously, with IL-1β acting as a pro-inflammatory cytokine and IL-10 thought to be mainly anti-inflammatory. However, siRNA-mediated TLR2 knockdown did not inhibit the induction of IL-1β to the same degree as its effect on IL-10, demonstrating the importance of pro-inflammatory mediators/receptors in the response to *F. solu* invasion [72].

### 3.2. Sterile Corneal Inflammation (Non-Infectious)

The previous sections provided a description of microorganisms that can cause innate immune system activation and inflammation (infectious keratitis), particularly when the corneal epithelial barrier is compromised, i.e., wounded. Additionally summarized were results suggesting that these processes provide a benefit in terms of clearing the microbial agents and preventing the significant potential visual morbidities that can accompany infection. Although the beneficial effects of the innate immune system on counteracting infections is obvious, there is evidence to suggest that the innate immune response may be excessive in terms of what is needed under most conditions. This overreaction by the innate immune system occurs presumably because during the course of evolution, the risk of adverse outcomes from infections facilitated by corneal wounding was a greater threat to vision, and thus survival, than a slightly delayed corneal wound healing response. The excessive activation of the immune system is even more apparent in non-infectious keratitis, which is characterized by inflammation in the absence of microbial infection and can be due to trauma, chemical burns, or autoimmune disorders. Nevertheless, there are indications in the literature that some inflammation is “good” and can promote wound healing [73]. Indeed, Eslani et al. [74] observed accelerated corneal wound healing upon treatment of mice with TLR4 agonists and inhibition with TLR4 antagonists. Thus, early TLR4 stimulation leads to an acute inflammatory response that promotes initial corneal epithelial wound healing, with inhibition of this early TLR4 activation resulting in delayed healing, due to decreases in cell proliferation and migration [74]. However, excessive and/or chronic inflammation is generally thought to be detrimental to wound healing, for instance, in non-healing (chronic) skin wounds, which are characterized by chronic inflammation [75,76].

Evidence in the literature suggests that non-microbially mediated inflammation, or sterile inflammation, can be the result of the release of endogenous molecules by endangered or damaged cells. These endogenous DAMPs have been found to be able to activate pattern recognition receptors similarly to the PAMPs. The ability of DAMPs acting through the innate immune system to promote sterile corneal inflammation has been demonstrated by Prockop and colleagues [77]. These scientists report that in a rodent model of sterile inflammation, the heat shock protein (HSP), heat shock protein B4 (HSPB4), released from corneal keratocytes exposed to damaged corneal epithelial cells, serves as a DAMP [30] to activate TLR2 on corneal macrophages. This activation stimulates inflammatory mediator expression and promotes neutrophil infiltration, in particular the later, sustained phase of the infiltration of neutrophils into the cornea upon wounding (the early phase is mediated by substance P released by neurons innervating the cornea) [77], through the nuclear factor kappa-light-chain-enhancer of activated B cells (NFκB) pathway. Inhibition of the HSPB4/TLR2/NFκB axis, in turn, can reduce neutrophil invasion into the cornea following injury, thus helping to preserve corneal clarity [77]. Thus, inhibition of HSPB4-induced TLR2 activation by the anti-inflammatory protein TSG decreases cytokine and chemokine production in the cornea to promote wound healing and inhibit corneal opacity following a sterile injury [78]. Other molecules that can serve as DAMPs, including β-defensin-2 and S100A proteins, may also be increased in the cornea and contribute to tissue inflammation [20,30,79]. These findings suggest that antagonists of DAMPs like HSPB4 and β-defensin-2 could serve a protective role to reduce chronic sterile inflammation and restore and maintain the structural integrity of the corneal epithelium.

Another study examined sterile corneal inflammation in relation to the NLRP3 inflammasome and its pro-inflammatory effects. In mice that were genetically engineered to ablate the gene encoding NLRP3, diminished neutrophil infiltration and reduced MMP9 and IL-1β expression following alkali burn injury were observed in NLRP3 knockout mice versus the wild-type mice. MMP9, a compound released in response to IL-1β via the NFκB and activator protein-1 (AP-1) pathways, is responsible for corneal neovascularization and inflammation [80]. NLRP3 increases corneal opacity as well as the levels of inflammatory cytokines, such as 1L-1β, following an alkali burn, and innate immunity was clearly activated in this case. However, treatment with NLRP3 inhibitors, such as sodium butyrate, β-hydroxybutyric acid and dexamethasone, results in a decrease in inflammatory cytokines and an improvement of corneal transparency [81]. Thus, the induction of proinflammatory cytokines, through activation of PRRs like the TLRs, can disturb the corneal epithelial structure, although, as noted previously, this innate immune system activation can also provide protection against and/or help to resolve microbial infection. Likewise, excessive stimulation of the innate immune system can enhance the severity of corneal disease secondary to increased inflammation. Activation of TLR2, TLR4, and TLR9 upregulates expression of chemokines through the MyD88 pathway, resulting in increased corneal haze and thickness, which can lead to visual impairment [82]. With chronic conditions like dry eye disease, TLR4 activation through LPS stimulation also leads to a more severe disease manifestation resulting from the increased production of pro-inflammatory cytokines such as IL-1β and CXCL10 in the cornea [83].

## 4. Diabetes and Corneal Wound Healing

Although the cornea normally heals rapidly, in some individuals this process occurs more slowly or not at all. Diabetes, in particular, can significantly delay corneal epithelial wound healing [84]. Up to 70% of diabetic patients examined display corneal problems [11], and there are over 30 million Americans with diabetes [85]. Diabetes also predisposes individuals to dry eye syndrome, as well as recurring corneal erosions and persistent corneal epithelial defects [84].

Treating corneal injuries in patients with diabetes can be clinically challenging since in addition to delayed healing, the damage is often accompanied by pain, neurotrophic keratitis, and recurrent erosions, as well as the possible superimposition of infection. The corneal epithelium in diabetic individuals is often thicker and stiffer [84,86], and the collagen IV anchoring fibrils are also abnormally shallow [87], which may result in an epithelium with reduced adhesion at risk of erosion as well as impaired barrier function [88]. Diabetes also affects the stroma, with glycation of stromal collagen resulting in aggregation and irregular cross-liking of fibrils, which reduces corneal clarity [89]. In the corneal endothelium, diabetes is associated with decreased endothelial cell density and function [90,91,92]; since the endothelium is critical for corneal dehydration, these changes can increase the risk of corneal edema, which also decreases corneal clarity. Finally, diabetes often induces corneal neuropathy as a result of glycation, oxidative and/or inflammatory damage, microvascular insult, and enzymatic actions on the nerve plexus that contribute to reduced nerve density, which can in turn result in neurotrophic ulcers and contribute to slow healing [93,94,95,96]. Reduced corneal nerve fiber density also leads to decreased levels of nerve-derived factors that promote corneal wound healing, such as substance P [84].

In addition to these structural alterations, changes in the inflammatory response play a significant role in corneal morbidity from diabetes. Thus, diabetes is accompanied by increases in serum high mobility group box-1 (HMGB1) [97], an endogenous molecule known to function as a DAMP to activate TLRs [30], such as TLR4. Advanced glycation end products, formed as a result of hyperglycemia and the oxidative state observed in diabetes, are also reported to activate TLRs [30]. In addition, advanced glycation end products activate the receptor for advanced glycation end products (RAGE), which is upstream of NFκB and can also promote inflammation. Indeed, elevated proinflammatory cytokines have been reported in diabetic corneas [98]. Therefore, the impaired wound healing observed with diabetes may also be related to a chronic inflammatory state.

## 5. Anti-Inflammatory Actions of Phosphatidylglycerol

Inflammation serves an important function in the body by helping to initiate the body’s first defense, the innate immune system, to manage microbial infections. However, too much or uncontrolled inflammation can lead to the pathogenesis of multiple diseases [99]. Thus, there is a need for treatments that can be used to suppress the innate immune system when it becomes overactive. Surfactant allows normal lung function [100] by reducing surface tension and preventing collapse of the alveoli of the lung. Indeed, a lack of surfactant has been shown to result in lung dysfunction [101]. Surfactant has also been found to serve a secondary but important function to reduce inflammation in the lungs [100]. Surfactant is composed of a mixture of lipids and proteins, and some of the proteins and lipids in surfactant have been shown to have anti-inflammatory effects [102]. The anti-inflammatory effect of surfactant is crucial, because the lungs are constantly exposed to outside irritants that could trigger an unwanted or excessive immune response resulting in damage to the lungs [100]. One component of surfactant in particular, the phospholipid phosphatidylglycerol, has been shown to reduce inflammation in the lungs induced by LPS and some viral pathogens. Phosphatidylglycerol has also been shown to reduce inflammation in mouse skin and in a mouse model of psoriasis [31,103]. Furthermore, other negatively charged phospholipids, such as phosphatidylinositol (which is also present in surfactant) and phosphatidylserine, have also been shown to exhibit anti-inflammatory properties in some systems [102,104,105]. The anti-inflammatory effects of phosphatidylglycerol (and other anti-inflammatory phospholipids) and its interaction with the immune system are only now becoming better understood.

Certain phosphatidylglycerol species have been found to exert anti-inflammatory effects in various cell types and tissues. As mentioned above, phosphatidylglycerol is found in pulmonary surfactant in the lungs, with the main species in humans being palmitoyl, oleoylphosphatidylglycerol (POPG) [106]. Both POPG and dioleoylphosphatidylglycerol (DOPG) have been found to reduce lung inflammation, for instance, decreasing pulmonary TNFα secretion and/or the expression of phospholipase A_2_ (PLA_2_) in a dose-dependent manner [107]. DOPG has also been found to inhibit NFκB activation in the lung; since activated NFκB induces the expression of secretory PLA_2_ (sPLA_2_), which produces pro-inflammatory eicosanoids upon endotoxin stimulation of alveolar macrophages [106], the effect of DOPG is to suppress lung inflammation. DOPG also reduces TLR1/2-induced inflammatory mediator expression in skin keratinocytes; however, in the macrophage cell line RAW264.7, DOPG is able to inhibit the expression of some, but not all inflammatory mediators, although the reason for this disparity is unknown [108]. The phosphatidylglycerol species dimyristoylphosphatidylglycerol has also been found to inhibit LPS-induced production of TNFα and nitric oxide, as has the phospholipid phosphatidylinositol [102].

Phosphatidylglycerol has also been shown to inhibit sterile inflammation in response to DAMPs. DOPG and soy phosphatidylglycerol (a mixture of various phosphatidylglycerol species) have been found to inhibit inflammatory mediator expression induced as a result of TL2 and TLR4 activation by the antimicrobial peptide and DAMP S100A9 in skin epidermal keratinocytes [108], implying that phosphatidylglycerol’s anti-inflammatory effects occur more universally in the body and not only in the lungs. In a macrophage cell line DOPG is able to block S100A9-induced NFκB translocation into the nucleus, an indicator of this transcription factor’s activation, and a necessary step for its regulation of target genes [31]. PAMP-elicited inflammatory mediator expression in keratinocytes can also be alleviated by the application of DOPG or soy phosphatidylglycerol [108]. Multiple species of phosphatidylglycerol have also been shown to inhibit TLR4 activation induced by LPS [108], as well as TLR2 activation in response to DAMPs, as well as to PAMPs. For example, DOPG was found to reduce the activation of TLR1/2 in keratinocytes exposed to Pam_3_CSK_4_, a synthetic triacylated lipopeptide agonist of TLR1/2 [108]. In addition, the antimicrobial peptide DAMP β-defensin-2, expressed in epidermal keratinocytes and upregulated in the skin disease psoriasis [109], has been found to activate TLR2, and this effect is blocked by DOPG [31]. However, phosphatidylglycerol does not block all immune responses. DOPG has only a minimal effect to reduce the activation of NFκB in response to the TLR7/8 agonist resiquimod [31]; similarly, POPG was found to have no significant effect on flagellin-mediated TLR5 or CG-rich oligonucleotide (CpG)-mediated TLR9 activation [102]. These results indicate that DOPG does not inhibit NFκB directly or TLRs in general, implying that DOPG would not be globally immunosuppressive, as is seen with some anti-inflammatory therapies [31,108].

Phosphatidylglycerol has been found to inhibit TLR4 activation in more than one way. POPG was found to inhibit LPS-induced inflammation by directly binding to LPS [102]. In addition, as discussed below, POPG was found to bind to CD-14 and MD-2 [102], which are important TLR4-interacting proteins necessary for TLR4 activation [28,110]. POPG has also been found to exhibit antiviral properties; for example, it directly binds to respiratory syncytial virus (RSV) and prevents RSV infection in the lungs in vivo, with a reduction in viral load, immune cell infiltration and interferon-γ and surfactant protein-D (SP-D) levels, and decreases inflammation [111]. POPG has also been shown to affect influenza A virus (IAV) strains H3N2 and H1N1 by directly binding to them in a concentration saturable manner, but without acting as a virucidal agent [112]. POPG also prevents the lethality of this virus in mice and blocks the spread of secondary infection after establishment of a primary infection [113]. Perhaps more importantly, POPG also inhibits H1N1 infection in ferrets, a preferred animal model for studying influenza A [113]. It appears that one way in which phosphatidylglycerol inhibits inflammation may be by binding directly to PAMPs and certain viruses, thus preventing them from activating TLRs or infecting cells. Another mechanism may involve the TLR2 and TLR4 co-receptor CD-14 [27], which has been shown to bind phosphatidylglycerol (as has MD-2) [111]. Indeed, the ability of phosphatidylglycerol analogs to inhibit LPS-induced TLR4 activation correlates with their affinity for CD-14 [114], suggesting this protein as a likely phosphatidylglycerol target.

There are some effects of phosphatidylglycerol for which the mechanism of action is as yet unknown. Recently, DOPG was found to accelerate wound healing in the cornea in both wild-type mice and in mice with impaired corneal wound healing due to the ablation of the aquaporin-3 gene [1]; however, the mechanism of this enhancement is currently unknown. Phosphatidylglycerol supplementation was also shown to rescue impaired mitochondrial activity after exposure of RAW 264.7 (macrophage) cells to KLA [115], a synthetic analog of LPS. This phospholipid also protects retinal epithelial cells from apoptosis in response to a retinoid metabolite known to accumulate with age and associated with the development of age-related macular degeneration [116]. In addition, phosphatidylglycerol is known to activate protein kinases, such as protein kinase C (PKC)-βII and PKC-θ [117,118,119], and it seems possible that downstream signaling cascades from these kinases could exert some of the effects observed with phosphatidylglycerol treatment. Thus, further studies into the mechanism(s) of phosphatidylglycerol’s actions are clearly warranted.

## 6. Phosphatidylglycerol as a Potential Therapeutic Treatment

It seems likely that phosphatidylglycerol could be developed as a therapy to enhance corneal wound healing and suppress corneal inflammation. Indeed, synthetic surfactant containing phosphatidylglycerol is already approved by the Food and Drug Administration (FDA) as a treatment for respiratory distress syndrome in infants [120]. Furthermore, most phosphatidylglycerol species have been shown to have few or no toxic effects on various cell types [102,108,120]. Therefore, phosphatidylglycerol, a major lipid in surfactant, offers potential as a therapeutic treatment due to its anti-inflammatory effects. For example, DOPG applied topically ameliorates inflammatory psoriasis-like skin lesions in the imiquimod-induced mouse model of psoriasis [31]. DOPG has further been shown to reduce immune cell numbers in bronchoalveolar lavage fluid, as well as the inflammatory cytokine TNFα and sPLA2 in damaged lungs of piglets [121]. Furthermore, in this piglet model of lung injury, animals treated with DOPG exhibit reduced alveolar epithelial apoptosis and suppression of epithelial growth factors, e.g., amphiregulin and TGFβ1, that induce epithelial to mesenchymal transition, thereby leading to fibrosis [121]. Finally, as mentioned above, POPG, in addition to inhibiting lung inflammation, also inhibits infection with pH1N1 influenza A virus in ferrets [113].

**Figure 3:**
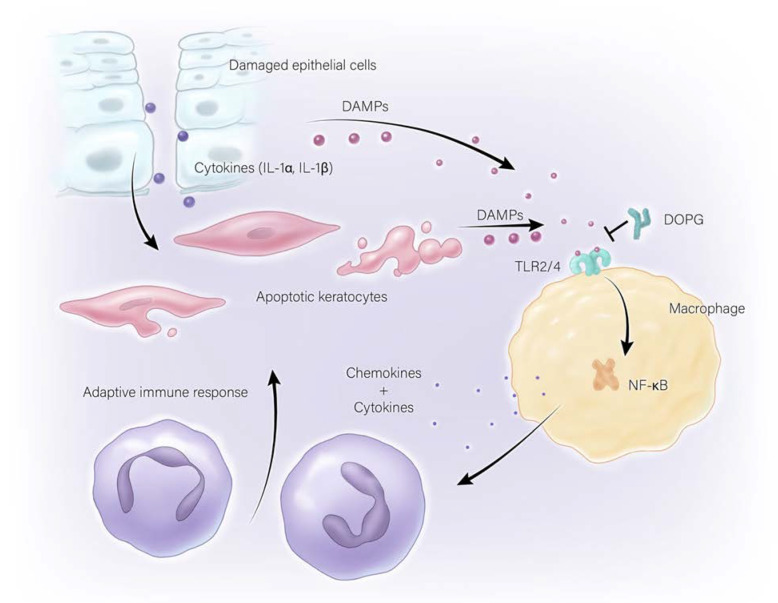
Innate Immune System Activation upon Corneal Wounding. As shown, corneal wounding results in the release of cytokines like interleukin-1 (IL-1), which can lead to keratocyte apoptosis and the release of endogenous molecules, called danger- or damage-associated molecular patterns (DAMPs); DAMPs can also be released from the injured corneal epithelial cells. DAMPs are able to activate pattern recognition receptors of the innate immune system such as toll-like receptors-2 and -4 (TLR2/4), which can function through the nuclear factor kappa-light-chain-enhancer of activated B cells (NFκB) pathway to induce the production and release of additional cytokines and chemokines that induce infiltration of innate and adaptive immune cells into the cornea. This immune cell recruitment can then lead to chronic sterile inflammation, which can in turn, impair vision. The phospholipid phosphatidylglycerol has been found to inhibit TLR2/4 activation by DAMPs and microbial components known as pathogen-associated molecular patterns. This effect, as well as its ability to enhance corneal wound healing, suggest the possibility that it might be useful as a therapy to reduce inflammation and improve corneal wound healing.

Due to the anti-inflammatory properties of phosphatidylglycerol, this phospholipid has potential as a treatment for multiple inflammatory health conditions, including lung inflammation caused by viral or bacterial infection, psoriasis in the skin, and especially wound healing and sterile inflammation in the cornea. Based on results collected to date, additional studies to completely define phosphatidylglycerol’s anti-inflammatory properties, as well as other potential therapeutic applications, certainly seem warranted. In addition, phosphatidylglycerol should be safe for use in the eye since a species of phosphatidylglycerol, dimyristoylphosphatidylglycerol, is already currently an *inactive* ingredient in Systane lubricant eye drops (please see the inactive ingredient list at the Systane website [122]). Dimyristoylphosphatidylglycerol has also been examined as a stabilizer of the tear film in contact lens wearers (Clinical Trial: ACTRN12613001323718) [123]. In addition, egg-derived phosphatidylglycerol is an *inactive* ingredient of Visudyne, an FDA-approved injectable drug used for photodynamic therapy for the treatment of age-related macular degeneration (please see the FDA website [124]). This current use of phosphatidylglycerol in approved applications, as well as the presence of phosphatidylglycerol in corneal epithelium [125], suggests that DOPG should be a useful therapy for the enhancement of wound healing and inhibition of inflammation in patients with corneal injuries. Indeed, adjustment of the phosphatidylglycerol dose should allow the induction of a small amount of initial inflammation, which appears to be necessary for proper rapid wound healing and/or clearance of any microorganisms, as discussed in previous sections, while suppressing excessive and/or chronic inflammation that damages tissue and exacerbates poor outcomes in corneal wound healing, as illustrated in Figure 3. Therefore, based on the data showing that a species of phosphatidylglycerol accelerates corneal wound healing in mice in vivo [1], as well as the wealth of evidence that excessive innate immune system activation and inflammation is harmful to the cornea (as reviewed here), it seems that phosphatidylglycerol should be examined for development as a potential corneal wound healing therapy.

## 7. Conclusions

Inflammation is produced by the immune system in response to injury in order to prevent or combat potential microbial invasion and clear microorganisms from the wound, as discussed in Section 3.1. However, experimental evidence suggests that this initial response may be sufficient to somewhat impair wound healing, since during the course of evolution, infection was likely a greater threat to organismal survival than was a slight delay in wound closure. Thus, inhibition of wounding-induced innate immune system activation and initial inflammation, through the various manipulations discussed in previous sections, improves corneal wound healing. Phosphatidylglycerol, in particular DOPG, also accelerates epithelial corneal wound healing [1], although the mechanism of its action is unknown. Phosphatidylglycerol also exerts anti-inflammatory effects as described in Section 6, suggesting that the ability of this phospholipid to inhibit PAMP- and DAMP-induced TLR activation and resultant inflammation might underlie its promotion of corneal wound healing. Finally, phosphatidylglycerol is already an ingredient in cosmetics, eye drops and medications, indicating its safety for human use. Therefore, phosphatidylglycerol could be a safe, inexpensive and efficacious therapy to improve corneal wound healing and suppress inflammation upon injury, even in individuals with impaired healing, such as those with diabetes.

## Data Availability

Not applicable.
